# Construction of bioactive nanocomposites from beta-sitosterol, fucoidan, chitosan, and silver nanoparticles for anticancer applications

**DOI:** 10.3389/fbioe.2025.1668888

**Published:** 2025-10-15

**Authors:** Ahmed A. Tayel, Fatma K. Elagezy, Asmaa Abdella, Hend A. Gad, Mohamed E. El-Hefnawy, Sultan Alhayyani, Shaymaa W. El-Far

**Affiliations:** ^1^ Department of Fish Processing and Biotechnology, Faculty of Aquatic and Fisheries Sciences, Kafrelsheikh University, Kafrelsheikh, Egypt; ^2^ Department of Industrial Biotechnology, Genetic Engineering and Biotechnology Research Institute, University of Sadat City, El-Sadat City, Egypt; ^3^ Department of Marine Chemistry, Faculty of Marine Sciences, King Abdulaziz University, Jeddah, Saudi Arabia; ^4^ Department of Chemistry, College of Sciences and Arts, King Abdulaziz University, Rabigh, Saudi Arabia; ^5^ Division of Pharmaceutical Microbiology, Department of Pharmaceutics and Industrial Pharmacy, College of Pharmacy, Taif University, Taif, Saudi Arabia

**Keywords:** anticancer activity, biopolymers nanoparticles, biosynthesis, natural products, bioactivity

## Abstract

**Introduction:**

Cancers (e.g., colon and skin cancers) are significant causes of mortality. We developed novel anticancer nanocomposites comprising natural bioactive compounds and metal nanoparticles, involving conjugation of beta-sitosterol (BSt) and fucoidan (Fu), for biogenic synthesis of silver nanoparticles (AgNPs), before conjugation with chitosan (CS), to generate potential innovative anticancerous nanocomposites.

**Methods:**

The fabricated Fu/BSt/AgNPs/CS nanocomposites were characterized for efficacy against colon cancer (HCT-116) and skin cancer (A375) cells. Electron microscopy (SEM and TEM), FTIR infrared spectroscopy, DLS analysis and UV-Vis spectroscopy, confirmed the synthesis and interactions within nanocomposites. The MTT assay and dual staining validated the potential nanocomposites’ actions as anticancerous materials, compared to “cisplatin”.

**Results and discussion:**

AgNPs had a mean diameter of 8.83 nm, whereas Fu/BSt/AgNPs/CS had 146.6 nm size. MTT assay revealed that IC_50_ of Fu/BSt/AgNPs and Fu/BSt/AgNPs/CS nanocomposites were 16.23, 12.75 mg/L against HCT-116 cells, and 34.81, 22.44 mg/L against A375 cells, respectively, which significantly exceeded the cisplatin IC_50_ (25.56 and 79.77, respectively). The cancer cells’ treatment with nanocomposites revealed significant apoptosis induction and cell growth inhibition. Ultra-structural imaging confirmed the nanocomposites’ ability to trigger cancer cells to undergo morphological alterations, destructions and collapse. Invented Fu/BSt/AgNPs/CS nanocomposites have great promises as safe and effective treatments for colon and skin cancers, generated from natural compounds.

## 1 Introduction

Cancer has historically been and continues to be the second leading cause of death worldwide, with millions of new cases annually (Nandi et al., 2024), making it a significant global public health concern (Siegel et al., 2022). Cancer is a medical condition that affects people all around the world and is distinguished by abnormal cell proliferation resulting from various changes in gene expression, including mutations in adult stem cells that can divide and multiply throughout postnatal life (Jiang et al., 2025). The estimated new cancer cases worldwide in 2015, 2020, 2022 and projection of 2025 are totally ∼17.5, 19.3, 20.0, and 21.1 million, including Colorectal (Colon and Rectum) Cancer with ∼1.7, 1.9, 2.1 and 2.3 million, whereas the new cases of skin cancer (Melanoma and Non-Melanoma) are 4.1, 5.2, 6.6 and 7.7 million, respectively (Siegel et al., 2022; Jiang et al., 2025). This class of diseases, which occurs in higher multicellular organs, is associated with alterations in the expression of multiple genes, leading to the disruption of normal programmed cell division and differentiation (Jiang et al., 2024). Ultimately, this leads to an imbalance in the control of cell division and death, which permits malignant cells to in-filtrate other organs and spread to far-off locations (Ji et al., 2021), promoting the growth of tumor cell mass (Islam et al., 2022; López Ruiz et al., 2020). The key managing approaches of cancers involve surgery, chemotherapy and radiotherapy, which could induce numerous serious side-effects, where their curative impacts are limited. Thus, the explorations of less toxic substances from natural origins are always research priorities of scientists. The potentiality of natural derivatives to target definite signaling pathways was proposed for inhibiting/delaying the carcinogenesis development at diverse phases; natural phytocompounds have also the ability to provide elevated functionality and specificity, lower cytotoxicity, and selective induction of apoptosis in cancerous cell (Jiang et al., 2025; Islam et al., 2022; Rytsyk et al., 2020).

The colon is an essential part of the human body as it is a lengthy tube that joins the rectum and small intestine.Its length can vary between 1 and 150 cm (5 and 6 ft) and together with the rectum, makes up the large intestine. This specialized organ is responsible for breaking down and storing waste before releasing it into the rectum for defecation to occur (Rytsyk et al., 2020). Colorectal cancer is the third leading cause of cancer anywhere and when detected early, it can be cured through surgery. However, advanced cases are often fatal, with liver metastases accounting for most of the deaths. Genetic mutations and oncogenes are recognized as aiding the development of colorectal cancer (Khan and Anwar, 2019). Additionally, it ranks as the second most frequent cause of cancer-related mortality among people, with increasing numbers worldwide each year. It also considered a common tumor in the gastrointestinal tract, responsible for 6% of new cancer diagnoses and 5.8% of cancer-related deaths worldwide each year (Zhang et al., 2022). Only 70% of colorectal tumors are operable when initially diagnosed, with a 75% chance of cure for those cases (Zhu et al., 2024). However, operable patients have a 25% probability of getting sick again. Nineteen percent of individuals have advanced illness at diagnosis. Every year, about 250,000 new instances of colon cancer are detected in Germany, accounting for 9% of all cancer cases. While it has been more prevalent in wealthy nations, it is also becoming increasingly prevalent in middle-class and low-income nations (Rytsyk et al., 2020; Zhang et al., 2022). Treatment for colon cancer usually involves surgery and chemotherapy, but these treatments can sometimes be ineffective. The treatments of colorectal cancers with natural and biological derivatives/compounds were suggested as potential effectual approaches fo managing these cells (Islam et al., 2022; Rytsyk et al., 2020; Zhang et al., 2022; Zhu et al., 2024).

The skin is the largest and the primary defense organ of body (Peate, 2021).Melanoma, a type of skin cancer, is becoming increasingly common and has a rapidly rising death rate (Liu et al., 2022). This aggressive disease can infiltrate lymph vessels and spread quickly, reaching other parts of the body through blood vessels. For example, melanocytes can enter lymph nodes and move to the lungs, posing a significant danger (Alves et al., 2024). The applications of natural derivatives, especially in nanoforms, are recurrently recommended for treating/managing the skin cancers (Peate, 2021; Liu et al., 2022; Alves et al., 2024).

Fucoidan (Fu), a polysaccharide comprising sulfated fucose residues, is abundant in marine life, particularly brown algae, where it is among the numerous bioactive molecules found (Díaz- et al., 2023). Numerous studies have been conducted on fucoidan’s anticancer effects. Basic research studies have demonstrated a several biological functions, including as antioxidant, anticancer, and anti-inflammatory anti-Helicobacter pylori, antiangiogenic, antiviral, antithrombotic, and anticoagulant effects (Zahariev et al., 2023). The sulfated nature of Fu advocated its role as antimicrobial, scavenging agent and anticancerous biopolymers, in addition to its potentiality for mediating diverse metals nanoparticles and conjugating them to provide more effectual bioactive nanocomposites (Rajeshkumar, 2017; Sacramento et al., 2022; Rajeshkumar et al., 2021; Li et al., 2022). Fucoidan can kill malignan-cies directly by causing apoptosis, cell cycle arrest, and other reactions. It can also kill cancer cells indirectly by enhancing the activity of natural killer cells, macrophages, and others (Rajeshkumar et al., 2021; Li et al., 2022; Hsiao et al., 2022; Namvar et al., 2013). Fucoidan’s potent biological activity, broad availability, low vulnerability to drug resistance, and low side effects make it a desirable choice for usage as a new an-ti-tumor agent or as an adjuvant in conjunction with current therapies (Zahariev et al., 2023; Sacramento et al., 2022).

Phytosterols (PS) include ß-sitosterol (BSt), a bioactive food component that may possess potential for cancer prevention and treatment by impacting various regulatory mechanisms. Studies have indicated that BSt, derived from plants, has demonstrated anticancer characteristics that protect against stomach, lung, colon, prostate, skin, and leukemia malignancies. Furthermore, ß-sitosterol has been found to have minimally harmful anti-inflammatory, anticancer, hepatic protecting, antioxidant, cardio protective and antidiabetic properties through pharmacological screening. It has also been observed to interfere with multiple cellular pathways of communication, involving invasion, survival, metastasis, angiogenesis, apoptosis, proliferation, and phase of cell. Furthermore, the compound has displayed antioxidant activity by scavenging free radicals 2,2-diphenylpicrylhydrazyl (DPPH) (Alvarez-Sala et al., 2019; Bao et al., 2022).

Natural polymers are regarded as eco-friendly alternatives and are extensively utilized across Because of their non-toxic, sustainable, and renewable qualities; they are used in the food, medicinal, related to farming, and ecology sectors (Yu et al., 2020). Chitosan (CS), a bioactive deacetylated form of chitin, has been effectively extracted from various sources, including crustacean waste, fungal mycelia, and insect exoskeletons (Aranaz et al., 2021). Chitosan nanoparticles (CS-NPs) have gained increasing attention for their potential uses in crops fertilization, disease prevention, preservation of the earth, medication encapsulation and delivery, and health protection (Jhaveri et al., 2021). Chitosan and its derivatives are recognized as promising natural polysaccharides with anticancer potential. Extensive efforts to discover effective anticancer agents from natural sources have led to growing interest in polysaccharides. The bioactive properties of CS have driven its widespread use in various biomedical applications, including antimicrobial formulations, tissue design, anticancer therapy, administration of medications, and dressings for wound healing (Alalawy et al., 2020).

The unique characteristics of AgNPs make them valuable in tissue engineering. Different biomaterials have been created for various purposes, particularly because of their diverse reactions, modes of action, biodegradability, and biocompatibility (Tayel et al., 2021). *In vitro* models are fast, convenient, cost-effective, and helpful for assessing cytotoxicity, genotoxicity, biocompatibility, and effectiveness and performance (Zhang et al., 2016). Numerous investigations reported the usages of natural components as anti-cancerous bases (Jiang et al., 2025; Islam et al., 2022), including β‐sitosterol (Nandi et al., 2024; Alvarez-Sala et al., 2019; Bao et al., 2022), AgNPs (López Ruiz et al., 2020; Rajeshkumar, 2017; Zhang et al., 2016) fucoidan (Zahariev et al., 2023; Rajeshkumar, 2017; Rajeshkumar et al., 2021; Hsiao et al., 2022) and chitosan (Yu et al., 2020; Aranaz et al., 2021; Jhaveri et al., 2021; Alalawy et al., 2020), involving their nanoparticles and nanocomposites. Perceived insufficiency and inconsistency were reported from such natural compounds, addressing the need for deep and detailed investigations to develop more effectual combinations from them (Jiang et al., 2025; Islam et al., 2022).

This study aims to prevent cancer and provide potential anticancer alternatives from natural sources by exploring dietary and natural biomolecules, as well as nanomaterials, with the potential to reduce cancer incidence and progression, offering promising substitutions to traditional chemotherapy. The aim was to develop formulations with strong anticancer effects that could contribute to effective cancer treatments with minimal side-effects. Nanocomposites combining beta-sitosterol, fucoidan, chitosan and silver nanoparticles are generated using biogenic and polyelectrolyte compositing approaches, characterized, and tested for their anticancer properties against skin and colon cancer cell lines.

## 2 Materials and methods

### 2.1 Fucoidan extraction

The fucoidan (Fu) extracting involved the algal material dehydration, grinding then sterol was used to extract lipids, phenols, and terpenes. Extraction with ethanol (99%) and water is used to remove lipids and polysaccharides, followed by the use of an ethanolic solution to extract aldehyde, mannitol, chlorophyll, proteins, polyphenols, and nucleic acids (Ptak et al., 2021). After extraction, the precipitate was washed and heated. A chemical extraction process was then used to extract crude fucoidan by using a 1:20 ratio of (seaweed: extracting liquid) and 0.1 M HCl (37%) solution, heated at 70 °C and centrifuged “SIGMA 2–16 KL centrifuge; Sigma Lab. GmbH, Germany” at 10.500 xg for 10 min. NaOH solution was added to reach pH 7.0, followed by purifying procedures, involved the drying and milling of alginate into a powder after being treated with weak HCl to create alginic acid. To precipitate Fu and eliminate salts and small particles, 3 vol of cold ethanol was added (Costa et al., 2021). Cetyltrimethylammonium bromide (CTAB; ≥96.0%, Molecular Weight:364.45 Da; Merck, Germany) can be added during the extraction process to facilitate DNA isolation and separation of proteins. FTIR analysis was then conducted to confirm the extraction of Fu (Zayed et al., 2020).

### 2.2 Preparation of fucoidan and beta-sitosterol (Fu/BSt) composite

The preparation of Fu/BSt composite involved the intermixing of equal volume and concentrations of Fu and BSt under stirring “AREX-6; VELP Scientific Srl., Italy”. BSt (e.g., 0.75 g) was dissolved in 100 mL of 90% ethanol at room temperature for 45 min, whereas the same concentration was made from Fu in deionized water. Finally, equal volume of the solutions was mixed and stirred (620 xg) for 140 min to synthesize Fu/BSt composite. The composite were then vacuum-dried and milled.

### 2.3 Synthesis of Fu/BSt/AgNPs/CS nanocomposite

To prepare silver nanoparticles (AgNPs), a 10 mM solution of AgNO_3_ (≥99.0% purity; Merck KGaA, Darmstadt, Germany) was prepared in deionized water, and from chitosan (molecular weight = 50–190 kDa; Deacetylation degree = 75–85%; viscosity = 20–300 cP; Merck KGaA, Darmstadt, Germany), chitosan solution of 1.0% concentration (w/v) was prepared in 1.5% acetic acid aqueous solution. The Fu/BSt solution was made (1.0%, w/v) in deionized water. For the synthesis of the nanocomposite, equal volumes of Fu/BSt and AgNO_3_ (1:1) solutions were mixed and stirred using a magnetic stirrer (620 xg) for 3 h without heating. The pH of solution was adjusted to 8.5 via NaOH solution (0.1 M) dropping. Subsequently, equal amounts (e.g., 20 mL) of chitosan solution were slowly dropped into stirred Fu/BSt/AgNPs solution to complete the synthesis of the Fu/BSt/AgNPs/CS nanocomposite.

### 2.4 Fu/BSt/AgNPs/CS nanocomposite characterization

#### 2.4.1 NCs’ optical characterization

Using a UV-Vis spectroscopic examination (UV-2450, Shimadzu, Japan) at ranges between 200 and 800 nm, the formation of the Fu/BSt/AgNPs/CS nanocomposite was confirmed, and the absorbance wavenumber (λ max) for solutions with molecules that work together was measured.

#### 2.4.2 Fourier transform infrared (FTIR) spectroscopy

Using an infrared spectrometer (J FTIR; JASCO FT-IR-360, Japan), FTIR analysis provided data on the biochemical bonding between Fu/BSt/AgNPs and CS as well as their interactions in the composite. The solutions were processed into a powder, and combined with KBr before analysis. After that, the assessed molecules’ infrared transmission patterns appeared across a wavenumber range of 450–4,000 cm−1.

#### 2.4.3 Particles’ size (Ps) and zeta (ζ) potential appraisal

Using DLS “dynamic light scattering” and photon correlation spectroscopy techniques, the Ps distribution and surface charges (ζ potential) of synthesized NPs/NCs were evaluated using the Zetasizer (MalvernTM, Malvern, United Kingdom).

#### 2.4.4 The electron microscopy imaging

Scanning electron microscopy (SEM) and transmission electron microscopy (TEM) were used to examine the distribution, particle size (Ps), and optical shape of particles. The SEM (IT100, JEOL, Japan) was employed for scanning imaging, and the accelerating voltage was set to 10 kV. The nanocomposite suspensions were sterilized, assigned to self-adhesive carbon discs, and covered with a palladium/gold coating using a Polaron Inc. E5100 II sputter coater (Hatfield, PA) with the aim to get them suitable for SEM analysis. A TEM microscope “JEOL JEM-100CX, Japan” was used to evaluate the aqueous dispersions of the nanocomposites at an applied voltage of 200 kV after they were drop-cast onto carbon-coated copper grids and allowed to air dry at their natural temperature.

### 2.5 Monitoring a biological activity of cancer therapy

#### 2.5.1 Lines of cancerous cells

Our source for the HCT 116 and A375 cell lines was the ATCC “American Type Culture Collection, Rockville, United States.” 10% fetal bovine serum (FBS; Sigma Chemical Co., MO, USA) was added to RPMI1640, also known as “Roswell Park Memorial Institute medium,” in which the cells were cultivated. The cells were kept in an incubator with 5% CO_2_ at 37 °C in monolayer form. To evaluate the antitumor activity, triplicated experiments were conducted, where cisplatin was employed as a control drug for comparison.

#### 2.5.2 Cytotoxicity assays

The cytotoxicity assessments were conducted in the GEBRI-USC “Genetic Engineering and Biotechnology Research Institute, University of Sadat City, Egypt”, by specialized technicians. Mitochondrial function and cell viability were assessed using the MTT assay (Forma and Bryś, 2021). In summary, 1.0 × 10^5^ cells per well were impregnated into a 96-wells plate, and the cells were incubated for 24 ± 1 h under the previously mentioned conditions (5% CO_2_ at 37 °C). Cell-containing wells were treated with Fu/BSt/AgNPs/CS (T1, T2, and T3) at varying concentrations (e.g., 0–100.0 μg/mL). Following treatment, MTT solution served for heating the cells; 5.0 mg/mL solution of MTT “3-(4,5 Dimethylthiazol-2-yl)-2,5-diphenyltetrazolium bromide” was put into the inoculating wells, and they were incubated for additional 4 h. After removing the media from the wells, 100 μL of DMSO was transferred per well. The color of wells contents was then assessed colorimetrically (at 570 nm), after 30 min of moderate vortexing (El-Sherbiny et al., 2023). The anticancer activity of Fu/BSt/AgNPs/CS nanocomposites (at IC_50_ concentration) against A375 cells were further tracked using dual staining “acridine orange/ethydium bromide staining” (Alalawy et al., 2020; El-Sherbiny et al., 2023). The A375 cells (∼6 × 10^4^ cells) were treated by IC_50_ of Fu/BSt/AgNPs/CS nanocomposites and incubated for 24 h, 48 h, in addition to control treatment (only media) in 5% CO_2_ humidified air at 37 °C. Cells were bathed after treatment with phosphate buffer, and then were dual stained for 15 min with 4 μg/mL from each of acridine orange and ethydium bromide in dark. The fluorescence microscope “Olympus BX51, Tokyo, Japan” worked for capturing cells images to emphasize the immergence of apoptosis signs, e.g., the cells/organelles with green-, orange-, or red-stains, within 20–25 min from staining.

### 2.6 Scanning electron microscopy (SEM) imaging

Electron microscopy, particularly SEM, is a crucial process for examining the morphological characteristics and surface features of cancer cells, enabling detailed visualization of organelles and other cellular structures. Tumor cells often exhibit abnormal ultrastructural protrusions and self-assembled surface features that are not detectable under light microscopy but can be revealed through SEM. These surface architectures are critical for cell migration and motility, and by highlighting interactions between cells and their microenvironment, they provide key insights into cancer biology. Furthermore, the treatment led to a notable rise in apoptotic cells, confirming the anticancer properties of the nanocomposite (Venkatesan et al., 2017).

### 2.7 Statistical analysis

The assessment of nanomaterials’ characteristics and their biological (anticancer) activity were conducted in triplicates. The three replicates’ means were computed with Microsoft excel 2010. The unpaired *t*-test and one-way ANOVA was carried out, and the SPSS software program “V 17.0, SPSS Inc., Chicago, IL” was used to assess the significance of the results at p < 0. 05.

## 3 Results and discussion

### 3.1 Visual and optical identification

Fu/BSt/AgNPs composite was initially negative in charge, but upon mixing with chitosan, which carries a positive charge, a new composite, Fu/BSt/AgNPs/CS, was formed. The synthesis of the Fu/BSt/AgNPs/CS nanocomposite involved four key stages: (1) formation of the Fu/BSt composite solution, (2) preparation of the silver solution, (3) Within 45 min of mixing the two solutions, the color shifted to black, further confirming AgNPs, and (4) conjugation with CS to achieve to verify the Fu/BSt/AgNPs/CS tiny particles’ biogenesis.

**FIGURE 1:**
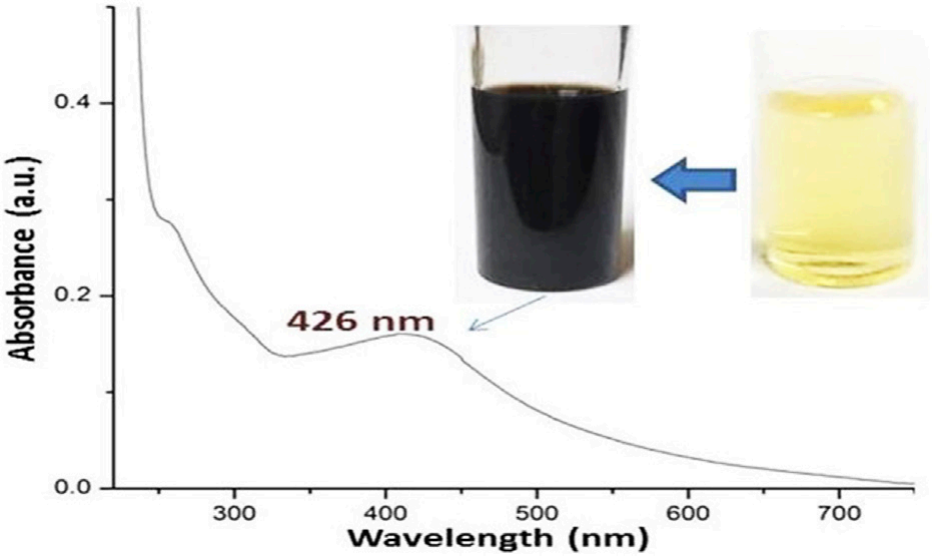
Visual aspects and UV-Vis spectrum of biosynthesized Fu/BSt/AgNPs nanocomposite.

Using Fu/BSt to verify the biosynthesis of AgNPs, UV-Vis spectroscopy was employed (Figure 1). Where the upper photos in Figure 1 reflected the color change (from bale yellow to dark brown) after the biosynthesis and reduction of AgNPs with Fu/BSt, the lower curve represents the UV spectrum of nanomaterial solution color, associated with AgNPs. The distinct surface plasmon resonance (SPR) peak of the AgNPs solution, with a λ max near 426 nm, indicated the successful biosynthesis of the nanocomposites (Yao et al., 2022).The color change of the biosynthesized AgNPs solution is typically associated with the size, dispersion, and morphology of the nanoparticles, as well as the reducing agents used (Menon et al., 2018).

#### 3.1.1 Infrared analysis of materials/nanocomposites

**FIGURE 2:**
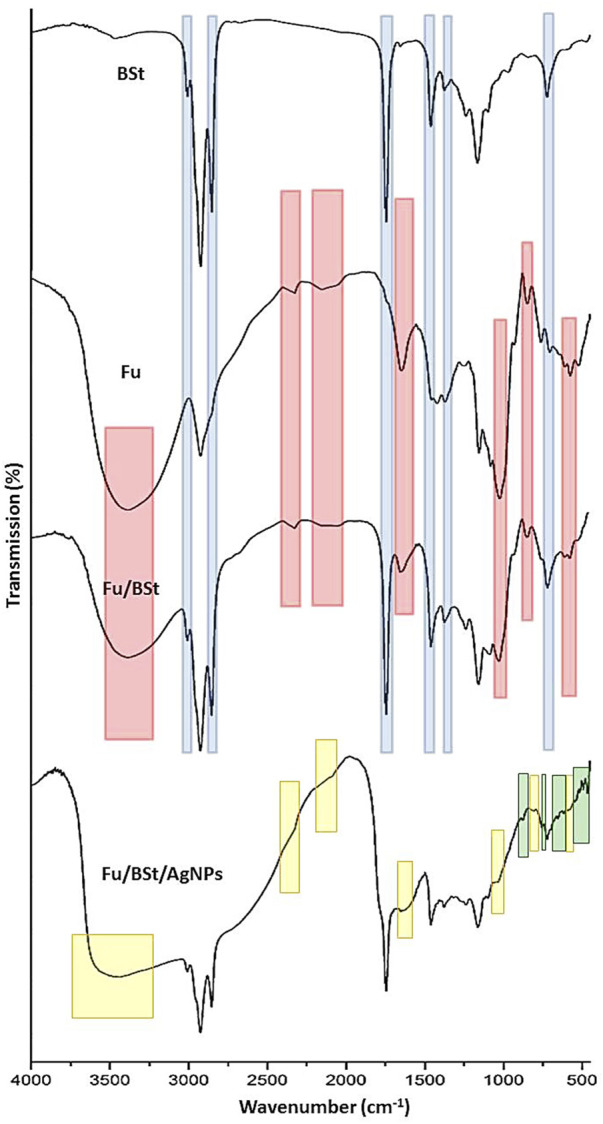
Infrared spectral analysis (FTIR) of fucoidan (Fu), beta sitosterol (BSt), fucoidan, beta sitosterol and silver nanoparticles Fu/BSt/AgNPs.

The FTIR analysis provides detailed insights into the molecular structure, functional groups, and interactions of the compounds under investigation (Figure 2). The IR spectra of the Fu, BSt, and their composites with AgNPs showed characteristic peaks that reflect the functional groups of the individual components, with some peak intensities altered as a result of interactions between them. The highlighted parts indicating the transferred, vanished or emerged bands after conjugation and interaction between conjugated materials.

For fucoidan (Fu), the key IR bands in this spectrum are detected at 1,556.6 cm^−1^, peaks representing groups of sulfides were detected, and at 1,038.2 cm^−1^ and 1,039.7 cm^−1^, they are ascribed to the extension motion of the C‒O‒S the central C4 locations with sulfate replacements. Peak number two at 1,558.8 cm^−1^ is associated with C‒H stretching of the pyrenoid ring, as well as the C-6 group of fructose and galactose (Figure 2). The asymmetric stretching of the sulfate group (O=S=O) is seen at 1,039.7 cm^−1^ (Yu et al., 2024). Additionally, while the band at 3,000 cm^−1^ represents C‒H stretching from the pyrenoid ring, a large peak focused at 3,433 cm^−1^ represents the bonded O‒H stretching vibration, and stretching of the glycosidic links from C to O to C. Peaks at 1750 cm^−1^ and 1,540 cm^−1^ are associated with the stretching vibrations of sulfate esters. The sulfide group’s C‒O‒S bending vibration is responsible for the band at 1,300 cm^−1^, while the bands at 590 cm^−1^ indicate the asymmetric and symmetric O=S=O deformation of the sulfate group. The FTIR spectrum demonstrated the success of the procedures used for getting rid of fucoidan from brown algae. Additionally, the asymmetric carboxylate O‒C‒O contraction is represented as a peak at 2,200 cm^−1^ (Narayani et al., 2019).

For beta-sitosterol (BSt), the infrared spectra showed notable absorbance peaks at 1749.9 cm−1 (C=C expanding), 2,959 cm^−1^ and 2,868.72 cm^−1^ (CH3), 2,868.72 cm^−1^ and 2,852.27 cm^−1^ (CH2), and 3,600 cm^−1^ (OH). Of the unconjugated olefin), and 1700 cm^−1^ (C‒OH deformation, associated with O‒C‒O symmetric stretching) Figure 2-BSt). Other notable bands include those for the secondary alcohol (C-OH) at 1,100 cm^−1^, the gem-dimethyl group (-CH(CH3)2) at 1,390 cm^−1^, and the cyclic methylene groups (CH2) at 1,484.5 cm^−1^ (Ododo et al., 2016).

According to the interaction between fucoidan and beta-sitosterol, the FTIR spectrum of the composite showed key adsorption features (Figure 2-Fu/BSt). A peak at 1,086 cm^−1^ was ascribed to the expanding of the C‒H‒O bond in alcohol and phenol compounds. The alcohol component’s C‒OH stretching vibrations Peaks at 1,086 cm^−1^ and 1,049 cm^−1^ confirmed the extract and nano-carrier, accordingly. Around 666 cm^−1^ and 881 cm^−1^, the bending frequencies linked to the C–H bonds in C–C–H classes were detected.

The FTIR spectrum of the Fu/BSt/AgNPs composite revealed distinct peaks at O-H and -NH_2_ stretching (3,359.22 cm^‒1^), C-H stretching (2,927.92 cm^‒1^), and -C-H stretches (2,838.25 cm^‒1^) (Figure 2-Fu/BSt/AgNPs). Additional noteworthy peaks are as follows: CH stretch (2,736.18 cm^−1^), C=O stretching (1,647.49 cm^−1^), ‒CONH2 (1,588.05 cm^−1^), OH bending (1,375.44 cm^−1^), C-N stretch (1,149.10 cm^−1^), C‒O stretching (1,079.13 cm^−1^), and 893.01 cm^−1^ (saccharide ring vibration) (Ata et al., 2025). The FTIR spectrum of the Fu/BSt/AgNPs nanocomposite further confirms the successful formation of the nanocomposite through characteristic shifts and the appearance of new peaks. After the synthesis of Fu/BSt/AgNPs, several changes in the spectra were observed. Notably, the disappearance of certain peaks, such as those related to the functional groups of fucoidan and beta-sitosterol (highlighted in yellow), suggests that some bonds were disrupted or altered during the nanoparticle synthesis process. This indicates that the silver nanoparticles are interacting with the biomolecules, leading to a modification of the original functional groups. Moreover, the emergence of new peaks (highlighted in green) in the Fu/BSt/AgNPs spectrum suggests the creation of fresh chemical connections between the proteins and the silver nanoparticles. These new peaks are indicative of the successful conjugation of AgNPs with the composite, confirming the formation of a stable nanocomposite material. The changes in the FTIR profile, including the shift in peak positions and the appearance of new absorption bands, strongly support the conclusion that the AgNPs are effectively integrated into the Fu/BSt matrix, forming a well-structured nanocomposite.

The highlighted zones in Fu/BSt spectrum are transferred from Fu (red zones) and from BSt (blue zones), whereas the highlighted parts in Fu/BSt/AgNPs with yellow indicated the vanished/disappeared peaks and the green zones are the emerged peaks after AgNPs biosynthesis and interaction with Fu/BSt.

### 3.2 Particles’ sizes and zeta (ζ) potential of fabricated nanomaterials

The DLS technique (Table 1) was used to demonstrate the diameters (Ds) and surface charges (ζ potential) of nanomaterials. It demonstates that each of the AgNPs, Fu/BSt, CS, and Fu/BSt/AgNPs/CS composite were negatively and positively charged as follow, - 23.45, - 26.18, +34.96, and +24.52 mV, respectively. As the higher values (>30 mV) results in higher NPs stability and dispersion, the DLS and TEM results matched for the screened nanoparticles (Figure 1), and their potentials might result in higher stabilities for manufactured nanocomposites (Khan and Anwar, 2019; Hou et al., 2022). The TEM imaging of the produced nanoparticles demonstrated their stability and good dispersion (Figure 1). AgNPs’ mean diameter was 7.23 nm, demonstrating the potent reduction activities of Fu/BSt that produced such small sizes. The mean Ds of Fu/BSt/AgNPs/CS composites (130.61 nm) was slightly greater than the mean Ds of individual Fu/BSt and AgNPs, which suggests the encapsulation of AgNPs within Fu/BSt particles (as earlier appointed in Figure 1).

**TABLE 1:** Particles’ sizes and charges (zeta potential) of fabricated nanomaterials.

Particles	Size range (nm)	Average size (nm)	Charge (mV)
AgNPs	1.41–33.42	7.23	−23.45
Fu/BSt	>1,000	>1,000	−26.18
CS	>1,000	>1,000	+34.96
Fu/BSt/AgNPs/CS	65.73–286.34	130.61	+24.52

### 3.3 The scanning electron microscope

**FIGURE 3:**
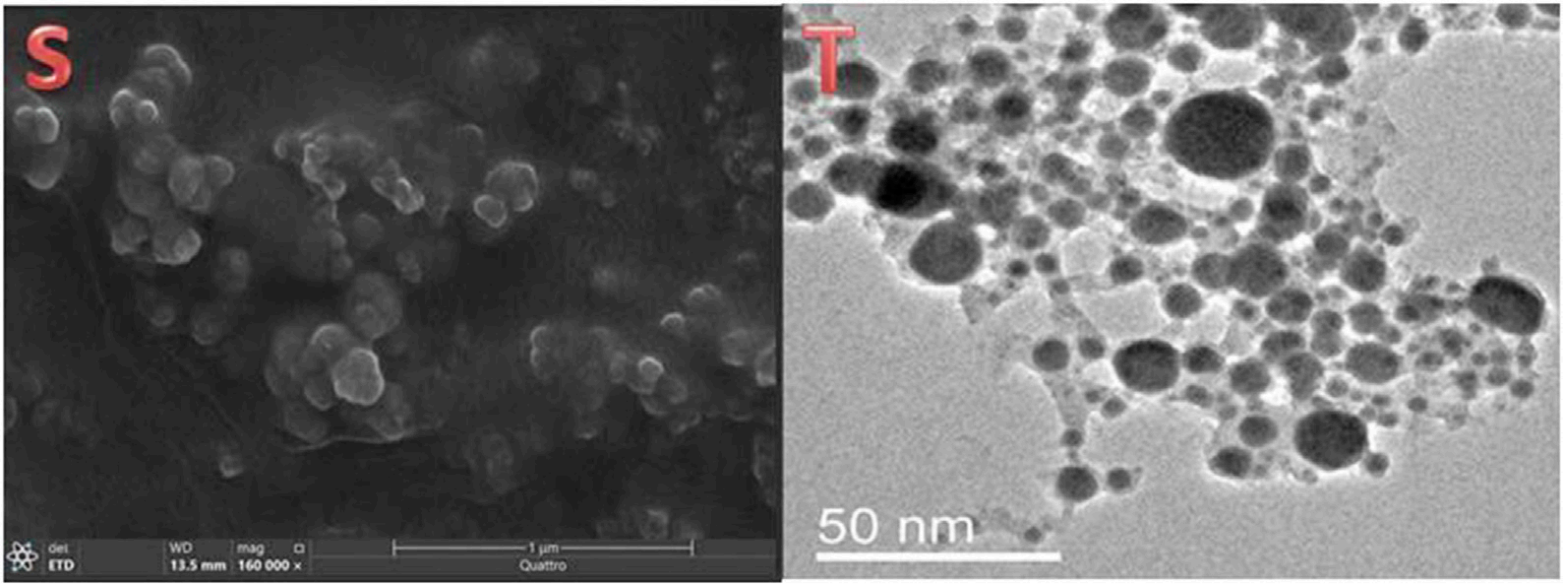
Electron microscopy imaging including scanning feature of Fu/BSt/AgNPs/CS **(S)** and transmission microscopy image of biosynthesized AgNPs **(T)**.

The SEM and TEM micrograph clearly demonstrate that the material exhibits a uniform dispersion of fine particles, with sizes typically in the nanoscale range (Figure 3). The images also reveal the nearly spherical shape and high resolution of the nanocomposite (Hou et al., 2022).

Imaging of the nanocomposites using SEM (Figure 3S) demonstrates the semi-spherical form of the granules, dispersion, and Ps distribution with an approximate size of 146.6 ± 4.32 nm. The successful formation of NCs confirmed the effectiveness of the newly employed synthesis procedure and may promote the use of this technique to create more NCs from other biological polymers. The primary factor suggested that the strong electrostatic interaction between the charged negative chitosan (CS) and the strongly charged CS is necessary for the efficient synthesis of the present NCs. Fu/BSt/AgNPs, which has been previously observed in other NCs formed through CS combining with biopolymers that are charged to the negative, such as the carrageenan and alginate (El-Sherbiny et al., 2022). The differences in charges between the positive CS and other negatively charged polymers enabled stable NCs formation with improved synergistic biological activity (El-Sherbiny et al., 2022). The size and morphology of AgNPs was captured through TEM analysis (Figure 3T), which appointed the mean diameter of 8.83 ± 1.51 nm of the particles.

### 3.4 *In vitro* anticancer biocidal activities of Fu/BSt/AgNPs/CS

The anticancer activity of the nanocomposite against HCT 116 and A375 cells was compared to cisplatin, a standard chemotherapeutic agent. The full IC_50_ values for all tested compounds across all cancer cell lines are presented in Table 2. The Fu/BSt/AgNPs/CS composite demonstrated the most effective anticancer effect, as demonstrated by the two cancer cell lines’ lowest IC50 values (e.g., 12.75 and 22.44 mg/L, respectively). The IC_50_ values against HCT 116 and A375 cells were 16.23 and 34.81 mg/L for Fu/BSt/AgNPs, 27.16 and 90.28 mg/L for BSt/AgNPs, 80.61 and 112.76 for BSt, respectively.

**TABLE 2:** Anticancer fucoidan (Fu), betasitosterol (BSt), fucoidan with beta sitosterol and silver nanoparticles, and their nanoconjugation with chitosan (Fu/BSt/AgNPs/CS).

Anticancer agent	IC_50_ (mg/L)^a^	
	HCT 116	A375
Fu	>200^a^	>200^a^
BSt	80.61 ± 5.67^b#^	112.76 ± 8.42^b^^
BSt/AgNPs	27.16 ± 2.18^c#^	90.28 ± 3.85^c^^
Fu/BSt/AgNPs	16.23 ± 3.05^f#^	34.81 ± 4.41^f#^
Fu/BSt/AgNPs/CS	12.75 ± 0.83^days#^	22.44 ± 1.39^days^^
Cisplatin	25.56 ± 1.62^c#^	79.77 ± 3.27^c^^

^a^ Dissimilar superscript symbols (within row) and letters (within column) indicate significant difference at p ≤ 0.05.

Interestingly, the cancer-fighting properties of the Fu/BSt/AgNPs/CS nanocomposite surpassed that of cisplatin (with IC_50_ of 25.56 and 79.77 mg/L against HCT 116 and A375 cells, respectively).

**FIGURE 4:**
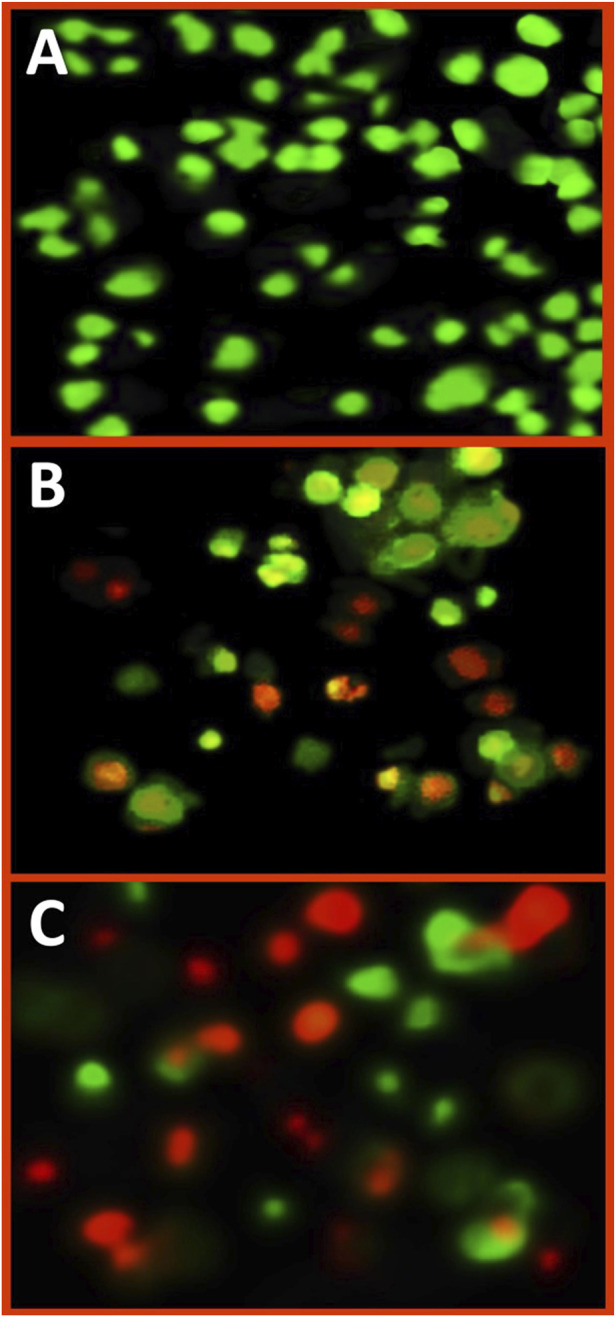
Fluorescent imaging of treated A375 cancer cells with IC_50_ concentration of Fu/BSt/AgNPs/CS nanocomposites, using dual staining with acridine orange/ethydium bromide, including control cells **(A)**, exposed cells for 24 h **(B)** and or 48 h **(C)**.

In contrast, the bare biopolymer CS exhibited lower biocidal activity compared to the Fu/BSt/AgNPs/CS nanocomposite against cancerous cells. The conjugation of nanometals with biopolymer coatings significantly reduced the potential toxicity to mammalian tissues while preserving or even enhancing the bioactivity against cancer cells (El-Sherbiny et al., 2023). The effect of the nanocomposite on HCT 116 colon cancer cells and A375 skin cancer cells, as demonstrated in Figure 4, revealed its cytotoxic potential. The synthesized nanocomposite notably inhibited cell proliferation. In terms of cell migration suppression, A375 and HCT 116 monolayers were exposed to different concentrations of the nanocomposite. After 12 h, treatment with 10, 15, and 20 mg/mL of the nanocomposite significantly reduced the migration rate of both cancer cell lines. After 48 h, the inhibition of cell migration for A375 and HCT 116 cells, respectively, in response to the nanocomposite treatment. Moreover, as the concentration of the nanocomposite increased, the migration inhibition also intensified, showing a clear dose-dependent effect.

### 3.5 Appraisal of cell apoptosis via dual staining and fluorescent imaging

The fluorescent imaging of treated A375 cells with Fu/BSt/AgNPs/CS nanocomposites (at IC_50_ concentration), using dual staining with acridine orange/ethydium bromide, could emphasize the impact of nanocomposite on the induction of apoptosis signs in cancerous cells (Figure 4). The color of control A375 cells was mostly green, without any reddish or orange spots (Figure 4A), which indicate the healthy and active conditions of cancer cells. After cells’ treatment with Fu/BSt/AgNPs/CS nanocomposites for 24 h, notable appearance of orange nuclei in several cells was detected with other cells that turned to reddish, which indicate cells death/apoptosis (Figure 4B). After 48 h of cells’ treatment, the quantity of apoptotic cells (reddish and reddish-orange stained) greatly increased with massive proportions (Figure 4C), which associates with the signs of apoptotic cells appearance. The appearance of apoptosis signs in treated cancer cells with nanocomposites were formerly stated in former investigations to verify the potentiality of nanomaterials for triggering cells death (Alalawy et al., 2020; El-Sherbiny et al., 2023).

### 3.6 Evaluation of the cytotoxic impact of nanocomposites using SEM

SEM scans revealed that the nanocomposite particles were present on the surface of tumor cells, which would not be visible with conventional light microscopy and required the higher resolution of SEM for detailed observation. These surface structures are critical for understanding cancer biology, as they play vital roles in cell migration, apoptosis, and overall cell function. Furthermore, cells treated with the nanocomposite exhibited clear signs of collapse, including membrane perforations and vacuolization. These deformations, previously observed in cells treated with anticancer agents (Zhang et al., 2024), emphasizing the potential impact of the nanocomposites on cell morphology and function, particularly in cancer treatment.

**FIGURE 5:**
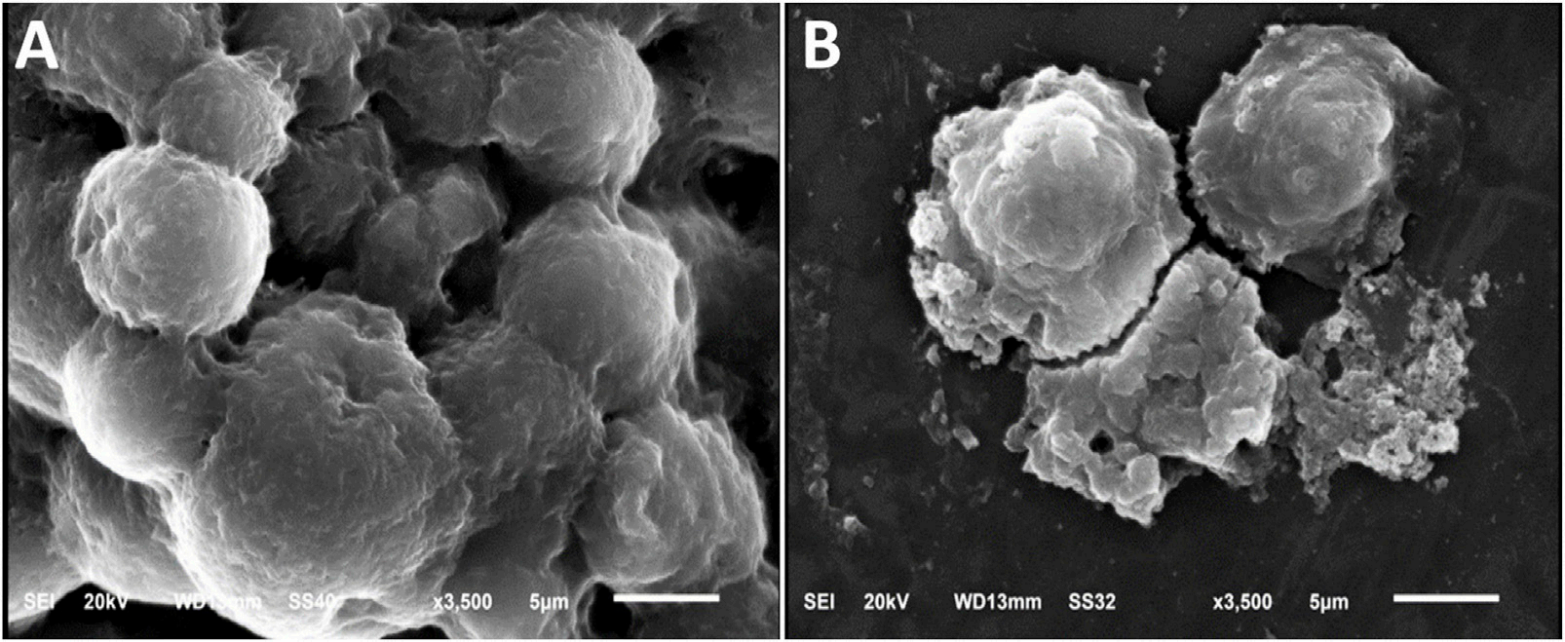
Morphological changes in HCT 116 cancerous cells before **(A)** and after **(B)** treatment with nanocomposite using scanning microscope.

The HCT 116 cells were chosen for SEM imaging because they were more sensitive to nanocomposites anticancerous action. After treating HCT 116 cells with 2 × IC_50_ of the Fu/BSt/AgNPs/CS nanocomposite, the anticancer potential of the composite was further examined using SEM imaging (Figure 5). SEM images revealed that control cells had a normal size and shape with intact structures, showing no abnormal features or distortions. In contrast, the treated cells displayed significant changes in cell morphology, including shrinkage, protrusion, and a reduction in cell density. These morphological alterations were accompanied by visible membrane perforations and vacuolization, indicating cell collapse. Such symmetric deformations and distortions are classic signs of cell death, suggesting that the Fu/BSt/AgNPs/CS nanocomposite effectively caused the cancer cells to alter morphologically.

## 4 Conclusion

The synthesized Fu/BSt/AgNPs nanocomposite conjugated with CS demonstrated significant promise as a natural and effective alternative for combating cancer, particularly in the suppression of colorectal (HCT-116) and skin (A375) malignant cells. The anticancer activity of the Fu/BSt/AgNPs/CS nanocomposite was notably superior to that of cisplatin, a conventional chemotherapy agent. Treatment with the nanocomposite led to cytotoxic effects, including apoptosis, reduced cell proliferation, and clear alterations in cell morphology, such as cell shrinkage, blebbing, and membrane perforation. SEM imaging revealed that these structural changes, such as vacuolization and membrane damage, are indicative of effective cell death, further supporting the anticancer potential of the nanocomposite. The bio-based components of the Fu/BSt/AgNPs/CS nanocomposite, combined with its potent anticancer properties, suggest a promising technique for controlling or preventing the growth of malignant cells. These results demonstrate the possibility of such nanocomposites as a viable and less toxic alternative for cancer therapy, with the ability to target tumor cells while minimizing damage to healthy tissues.

## Data Availability

The original contributions presented in the study are included in the article/supplementary material, further inquiries can be directed to the corresponding authors.

## References

[B1] Alalawy A. I. El Rabey H. A. Almutairi F. M. Tayel A. A. Al-Duais M. A. Zidan N. S. (2020). Effectual anticancer potentiality of loaded bee venom onto fungal chitosan nanoparticles. Int. J. Polym. Sci. 2020 (1), 1–9. 10.1155/2020/2785304

[B2] Alvarez-Sala A. Attanzio A. Tesoriere L. Garcia-Llatas G. Barberá R. Cilla A. (2019). Apoptotic effect of a phytosterol-ingredient and its main phytosterol (β-sitosterol) in human cancer cell lines. Int. J. food Sci. Nutr. 70 (3), 323–334. 10.1080/09637486.2018.1511689 30192685

[B3] Alves F. F. de Jesus L. C. Cristelli M. P. Enokihara M. M. E. S. Hirata S. H. Facina A. D. S. (2024). Metastasis of skin squamous cell carcinoma in kidney transplant recipients. Int. J. Dermatology 63 (5), 560–564. 10.1111/ijd.17029 38263692

[B4] Aranaz I. Alcántara A. R. Civera M. C. Arias C. Elorza B. Heras Caballero A. (2021). Chitosan: an overview of its properties and applications. Polymers 13 (19), 3256. 10.3390/polym13193256 34641071 PMC8512059

[B5] Ata T. E. Al-Ani I. Karameh N. Atta M. R. Dayyih W. A. (2025). Alectinib-loaded chitosan–alginate nanoparticles: a novel synthesis method with *in vitro* and *in vivo* evaluations. Pharmaceutics 17 (4), 492. 10.3390/pharmaceutics17040492 40284487 PMC12030190

[B6] Bao X. Zhang Y. Zhang H. Xia L. (2022). Molecular mechanism of β-sitosterol and its derivatives in tumor progression. Front. Oncol. 12, 926975. 10.3389/fonc.2022.926975 35756648 PMC9213880

[B7] Costa J. A. V. Lucas B. F. Alvarenga A. G. P. Moreira J. B. de Morais M. G. (2021). Microalgae polysaccharides: an overview of production, characterization, and potential applications. Polysaccharides 2 (4), 759–772. 10.3390/polysaccharides2040046

[B8] Díaz- Singh S. Chittasupho C. Prajapati B. G. Chandel A. S. (2023). Editorial: biodegradable polymeric materials in tissue engineering and their application in drug delivery. Front. Bioeng. Biotechnol. 11, 1296119. 10.3389/fbioe.2023.1296119 37840658 PMC10570791

[B9] El-Sherbiny M. M. Elekhtiar R. S. El-Hefnawy M. E. Mahrous H. Alhayyani S. Al-Goul S. T. (2022). Fabrication and assessment of potent anticancer nanoconjugates from chitosan nanoparticles, curcumin, and eugenol. Front. Bioeng. Biotechnol. 10, 1030936. 10.3389/fbioe.2022.1030936 36568301 PMC9773392

[B10] El-Sherbiny M. M. Orif M. I. El-Hefnawy M. E. Alhayyani S. Al-Goul S. T. Elekhtiar R. S. (2023). Fabrication of bioactive nanocomposites from chitosan, cress mucilage, and selenium nanoparticles with powerful antibacterial and anticancerous actions. Front. Microbiol. 14, 1210780. 10.3389/fmicb.2023.1210780 37547689 PMC10402636

[B11] Forma E. Bryś M. (2021). Anticancer activity of propolis and its compounds. Nutrients 13 (8), 2594. 10.3390/nu13082594 34444754 PMC8399583

[B12] Hou L. Zhong T. Cheng P. Long B. Shi L. Meng X. (2022). Self-assembled peptide-paclitaxel nanoparticles for enhancing therapeutic efficacy in colorectal cancer. Front. Bioeng. Biotechnol. 10. ‏ 10.3389/fbioe.2022.938662 36246349 PMC9554092

[B13] Hsiao W. C. Hong Y. H. Tsai Y. H. Lee Y. C. Patel A. K. Guo H. R. (2022). Extraction, biochemical characterization, and health effects of native and degraded fucoidans from Sargassum crispifolium. Polymers 14 (9), 1812. 10.3390/polym14091812 35566981 PMC9103907

[B14] Islam M. R. Akash S. Rahman M. M. Nowrin F. T. Akter T. Shohag S. (2022). Colon cancer and colorectal cancer: prevention and treatment by potential natural products. Chemico-biological Interact. 368, 110170. 10.1016/j.cbi.2022.110170 36202214

[B15] Jhaveri J. Raichura Z. Khan T. Momin M. Omri A. (2021). Chitosan nanoparticles-insight into properties, functionalization and applications in drug delivery and theranostics. Molecules 26 (2), 272. ‏. 10.3390/molecules26020272 33430478 PMC7827344

[B16] Ji Z. Tao S. Wang B. (2021). Editorial: artificial intelligence (AI) optimized systems modeling for the deeper understanding of human cancers. Front. Bioeng. Biotechnol. 9, 756314. 10.3389/fbioe.2021.756314 34708028 PMC8542901

[B17] Jiang Z. Wang X. Zhou Z. Peng L. Lin X. Luo X. (2024). Functional characterization of D-type cyclins involved in cell division in rice. BMC Plant Biol. 24 (1), 157. ‏ 10.1186/s12870-024-04828-9 38424498 PMC10905880

[B18] Jiang X. Yan-Ran W. Pin-Ru D. Shi-Yi Q. Haitao J. (2025). Organoids in cancer therapies: a comprehensive review. Front. Bioeng. Biotechnol. 13, 1607488. ‏ 10.3389/fbioe.2025.1607488 40766972 PMC12322668

[B19] Khan S. Anwar N. (2019). Highly porous ph‐responsive carboxymethyl chitosan‐grafted‐poly (acrylic acid) based smart hydrogels for 5‐fluorouracil controlled delivery and colon targeting. Int. J. Polym. Sci. 2019 (1), 1–15. 10.1155/2019/6579239

[B20] Li D. Zhang X. Chen X. Li W. (2022). Research progress and prospects for polymeric nanovesicles in anticancer drug delivery. Front. Bioeng. Biotechnol. 10, 850366. 10.3389/fbioe.2022.850366 35223804 PMC8874199

[B21] Liu Y. Li X. Liang A. (2022). Current research progress of local drug delivery systems based on biodegradable polymers in treating chronic osteomyelitis. Front. Bioeng. Biotechnol. 10, 1042128. 10.3389/fbioe.2022.1042128 36507256 PMC9729283

[B22] López Ruiz A. Bartomeu Garcia C. Navarro Gallón S. Webster T. J. (2020). Novel silver-platinum nanoparticles for anticancer and antimicrobial applications. Int. J. Nanomedicine Vol. 15, 169–179. 10.2147/ijn.s176737 32021172 PMC6970512

[B23] Menon S. Ks S. D. Santhiya R. Rajeshkumar S. Kumar V. (2018). Selenium nanoparticles: a potent chemotherapeutic agent and an elucidation of its mechanism. Colloids Surf. B. Biointerfaces. 170, 280–292. 10.1016/j.colsurfb.2018.06.006 29936381

[B24] Namvar F. Mohamad R. Baharara J. Zafar-Balanejad S. Fargahi F. Rahman H. S. (2013). Antioxidant, antiproliferative, and antiangiogenesis effects of polyphenol‐rich seaweed (Sargassum muticum). BioMed Res. Int. 2013 (1), 1–9. 10.1155/2013/604787 24078922 PMC3776361

[B25] Nandi S. Nag A. Khatua S. Sen S. Chakraborty N. Naskar A. (2024). Anticancer activity and other biomedical properties of β‐sitosterol: bridging phytochemistry and current pharmacological evidence for future translational approaches. Phytotherapy Res. 38 (2), 592–619. 10.1002/ptr.8061 37929761

[B26] Narayani S. S. Saravanan S. Ravindran J. Ramasamy M. S. Chitra J. (2019). *In vitro* anticancer activity of fucoidan extracted from Sargassum cinereum against Caco-2 cells. Int. J. Biol. Macromol. 138, 618–628. 10.1016/j.ijbiomac.2019.07.127 31344415

[B27] Ododo M. M. Choudhury M. K. Dekebo A. H. (2016). Structure elucidation of β-sitosterol with antibacterial activity from the root bark of Malva parviflora. SpringerPlus 5 (1), 1210–1211. ‏‏ 10.1186/s40064-016-2894-x 27516948 PMC4967061

[B28] Peate I. (2021). The skin: largest organ of the body. Br. J. Healthc. Assistants 15 (9), 446–451. 10.12968/bjha.2021.15.9.446

[B29] Ptak S. H. Sanchez L. Fretté X. Kurouski D. (2021). Complementarity of raman and infrared spectroscopy for rapid characterization of fucoidan extracts. Plant Methods 17, 130–10. ‏ 10.1186/s13007-021-00830-6 34930361 PMC8686358

[B30] Rajeshkumar S. (2017). Phytochemical constituents of fucoidan (*Padina tetrastromatica*) and its assisted AgNPs for enhanced antibacterial activity. IET nanobiotechnology 11 (3), 292–299. 10.1049/iet-nbt.2016.0099 28476987 PMC8676253

[B31] Rajeshkumar S. Sherif M. H. Malarkodi C. Ponnanikajamideen M. Arasu M. V. Al-Dhabi N. A. (2021). Cytotoxicity behaviour of response surface model optimized gold nanoparticles by utilizing fucoidan extracted from padina tetrastromatica. J. Mol. Struct. 1228, 129440. 10.1016/j.molstruc.2020.129440

[B32] Rytsyk O. Soroka Y. Shepet I. Vivchar Z. Andriichuk I. Lykhatskyi P. (2020). Experimental evaluation of the effectiveness of resveratrol as an antioxidant in colon cancer prevention. Nat. Product. Commun. 15. ‏ 10.1177/1934578x20932742

[B33] Sacramento M. M. Borges J. Correia F. J. Calado R. Rodrigues J. M. Patrício S. G. (2022). Green approaches for extraction, chemical modification and processing of marine polysaccharides for biomedical applications. Front. Bioeng. Biotechnol. 10. 10.3389/fbioe.2022.1041102 36568299 PMC9773402

[B34] Siegel R. L. Miller K. D. Fuchs H. E. Jemal A. (2022). Cancer statistics. CA a cancer J. Clin. 72 (1), 7–33. ‏ 10.3322/caac.21708 35020204

[B35] Tayel A. A. Ghanem R. A. Al-Saggaf M. S. Elebeedy D. Abd El Maksoud A. I. (2021). Application of Fish collagen‐nanochitosan‐henna extract composites for the control of skin pathogens and accelerating wound healing. Int. J. Polym. Sci. 2021 (1), 1–9. ‏ 10.1155/2021/1907914

[B36] Venkatesan J. Lee J. Y. Kang D. S. Anil S. Kim S. K. Shim M. S. (2017). Antimicrobial and anticancer activities of porous chitosan-alginate biosynthesized silver nanoparticles. Int. J. Biol. Macromol. 98, 515–525. ‏ 10.1016/j.ijbiomac.2017.01.120 28147234

[B37] Yao W. Qiu H. M. Cheong K. L. Zhong S. (2022). Advances in anti-cancer effects and underlying mechanisms of marine algae polysaccharides. Int. J. Biol. Macromol. 221, 472–485. 10.1016/j.ijbiomac.2022.09.055 36089081

[B38] Yu H. Wu W. Lin X. Feng Y. (2020). Polysaccharide-based nanomaterials for ocular drug delivery: a perspective. Front. Bioeng. Biotechnol. 8. ‏ 10.3389/fbioe.2020.601246 33363130 PMC7758246

[B39] Yu H. Zhang Q. Farooqi A. A. Wang J. Yue Y. Geng L. (2024). Opportunities and challenges of fucoidan for tumors therapy. Carbohydr. Polym. 324. ‏ 10.1016/j.carbpol.2023.121555 37985117

[B40] Zahariev N. Katsarov P. Lukova P. Pilicheva B. (2023). Novel fucoidan pharmaceutical formulations and their potential application in Oncology—A review. Polymers 15 (15), 3242. 10.3390/polym15153242 37571136 PMC10421178

[B41] Zayed A. El-Aasr M. Ibrahim A. R. S. Ulber R. (2020). Fucoidan characterization: determination of purity and physicochemical and chemical properties. Mar. drugs 18 (11), 571. 10.3390/md18110571 33228066 PMC7699409

[B42] Zhang X. F. Shen W. Gurunathan S. (2016). Silver nanoparticle-mediated cellular responses in various cell lines: an *in vitro* model. Int. J. Mol. Sci. 17 (10), 1603. 10.3390/ijms17101603 27669221 PMC5085636

[B43] Zhang Z. Lin S. Liu Z. Han J. Li J. Yu Y. (2022). Anticolon cancer targets and molecular mechanisms of tao‐he‐cheng‐qi formula. Evidence‐Based Complementary Altern. Med. 2022 (1), 1–22. ‏ 10.1155/2022/7998664 35479514 PMC9038428

[B44] Zhang T. Li X. Wu L. Su Y. Yang J. Zhu X. (2024). Enhanced cisplatin chemotherapy sensitivity by self-assembled nanoparticles with olaparib. Front. Bioeng. Biotechnol. 12, 1364975. 10.3389/fbioe.2024.1364975 38415186 PMC10898354

[B45] Zhu R. Yuan W. Xia A. Sun X. Yan W. Wu T. (2024). Inulin‐based nanoparticle modulates gut microbiota and immune microenvironment for improving colorectal cancer therapy. Adv. Funct. Mater. 34. 10.1002/adfm.202407685

